# Analysis of the Association of Mobile Phone Usage and Hearing Function in Young Adults

**DOI:** 10.7759/cureus.79403

**Published:** 2025-02-21

**Authors:** Ahmed I Haji, Haris Ejaz, Moaz O Omar, Mohamad B Takriti, Sareesh N Narayanan

**Affiliations:** 1 Internal Medicine, Ras Al Khaimah College of Medical Sciences, Ras Al Khaimah Medical and Health Sciences University, Ras Al Khaimah, ARE; 2 Anesthesia and Critical Care, Ras Al Khaimah College of Medical Sciences, Ras Al Khaimah Medical and Health Sciences University, Ras Al Khaimah, ARE; 3 Physiology, Ras Al Khaimah College of Medical Sciences, Ras Al Khaimah Medical and Health Sciences University, Ras Al Khaimah, ARE; 4 General Surgery, Ras Al Khaimah College of Medical Sciences, Ras Al Khaimah Medical and Health Sciences University, Ras Al Khaimah, ARE; 5 Physiology, School of Medicine and Dentistry, University of Central Lancashire, Preston, GBR

**Keywords:** 5g, hearing, mobile phone, radiofrequency electromagnetic radiation, young adults

## Abstract

Background: The health effects of radiofrequency electromagnetic radiation (RF-EMR), especially 4G and 5G, have been the subject of recent debate. The impact of this technology, particularly the nonthermal biological effects on humans and other animals has been largely ignored and not been comprehensively evaluated. Billions of people use this technology all over the globe today, and a significant percentage of them are in the adolescent age group. Therefore, a minor incidence of any adverse effect on health would be a major public health issue on a long-term basis.

Aim: The current study was designed to determine the association between long-, intermediate-, and/or short-term RF-EMR exposure from mobile phones on hearing ability in vulnerable populations like young adults.

Materials and methods: Seventy-eight young adults (aged 17-24 years) were recruited after obtaining their informed consent. First, a validated questionnaire was used to obtain the extent of phone usage among participants. They were then grouped as long-term, intermediate-term, and short-term users as per the criteria, and later their hearing ability was tested by pure tone audiometry.

Results: Among the 78 participants, 40 (51.28%) were males and 38 (48.71%) were females. Sixty-four (82.05%) of the participants responded that the right ear was their dominant ear, and for the remaining 14 (17.95%), it was the left ear. Forty-five participants (57.69%) use a 4G phone, while 32 (41.02%) use a 5G, and only one participant (1.28%) uses a 2G phone. Forty-five participants (57.69%) use the phone primarily for texting, while 33 (42.30%) use it to make calls. Fifty-four participants (69.23%) firmly attached the phone over their ears while making/receiving a call, but 24 (30.76%) did not. Fifty-nine participants (75.64%) did not feel any discomfort, but 11 (14.10%) felt a headache, one (1.28%) felt nausea, and seven (8.97%) experienced tinnitus during a call. In audiometry, mild to moderate hearing loss was evident in all three groups of participants at frequencies 250 Hz, 500 Hz, and 1000 Hz. The hearing loss was evident in individuals who have used their phones for more than 30 min/day for five years continuously compared to individuals who have used their phones 30 min/day for four years and/or 30 min/day for less than three years. This pattern was similar in both the right and left ears. Ear dominance did not play a significant role in influencing hearing loss in participants. However, significant hearing loss was found in 4G phone users compared to 5G phone users at 250 Hz and 500 Hz but not with frequencies between 1000 Hz and 8000 Hz particularly in the left ear. Moreover, irrespective of the participant's per-day average call duration, the hearing loss was evident at frequencies 250-1000Hz in both the left and right ears compared to other frequencies.

Conclusion: Long-term mobile phone use led to mild to moderate hearing loss, at frequencies 250 Hz, 500 Hz, and 1000 Hz. Ear dominance did not play a significant role in influencing hearing loss in participants. However, significant hearing loss was found in 4G phone users compared to 5G phone users at 250 Hz and 500 Hz but not with other frequencies. Further research is required to validate these results and understand RF-EMR effects on hearing in vulnerable populations like young adults.

## Introduction

Over three decades, mobile phone technology has influenced all facets of human life [1]. The objective of this technology is to assist people in communicating with each other at any place and time. While the world sees the tremendous growth of this technology, the health effects of radiofrequency electromagnetic radiation (RF-EMR), especially 5G emerged as a topic of recent debate. The usefulness of this technology is indisputable in this modern era as several lives are saved in cases of emergency. However, the impact of this technology, particularly the nonthermal biological effects on humans and other animals has been largely ignored and not been comprehensively evaluated [2]. The effect of RF-EMR from mobile phones and other wireless devices on human well-being and health is a thrust area of research over the globe. The primary reason for this is the exponential growth of mobile phone users worldwide in recent years [3]. In several countries, about half of the population uses this technology for communication purposes and is expected to increase rapidly. Globally, billions use mobile phone technology, and a significant percentage of them are adolescents. As a result, a small occurrence of any negative impact on health would be a major public health issue on a long-term basis. It is seen that not only the number of calls per day but also the length of each call plays a significant role in enhancing health-related risks in humans [4].

RF-EMR emitted by mobile phones is nonionizing, and it is absorbed by the brain when the phone is firmly placed over the head while talking. This increases the metabolism in brain regions closest to the antenna during acute mobile phone exposure suggesting that brain absorption of RF-EMR may enhance the excitability of brain tissue [5]. Although in 2011 the International Agency for Research on Cancer (IARC) classified mobile phone radiation as possibly carcinogenic to humans (which means, there could be some risk of carcinogenicity), they warrant additional research on long-term heavy users of this technology [6].

Although different frequencies of RF-EMR are extensively used in mobile telecommunications, the long-term cumulative effects that they may have on various organs have not been evaluated well [7]. When an individual uses the mobile phone to make a call, the ear remains closest to the phone and is the direct recipient of the noise, thermal energy, and EMR waves emitted by the phone. There is a concern that this may affect the sensitive outer hair cells in the organ of Corti [8]. Additionally, the long-term effects of RF-EMR on the functioning of outer and inner hair cells have also not been reported [7]. Some researchers have reported adverse health effects of RF-EMR such as changes in brain activity [9], reaction time (auditory reflex) [8], and changes in sleep patterns [10]. Despite all of this evidence, only a few reports have analyzed the impact of RF-EMR on the auditory system [11,12]. Another report suggests that RF-EMR exposure exceeding one hour/day may result in high-frequency hearing loss, and this was not necessarily induced by high volume which sometimes is a confounding factor in these types of studies [13]. The research data that assess the association between mobile phone use and hearing ability in humans are largely contradictory and inconclusive, which warrants further investigation. Moreover, the data on the association between long-term mobile phone use and hearing ability in the young adolescent population is also scanty. Hence, the current study was designed to determine the association between long-, intermediate-, and/or short-term RF-EMR exposure from mobile phones on hearing ability in vulnerable populations like young adults.

## Materials and methods

This cross-sectional study was conducted among 78 students (aged 17-24 years) of Ras Al Khaimah Medical and Health Sciences University with their informed consent. The institutional ethics committee approved all the procedures used in this study (REC-035-2021/22-UG-M). Healthy young adult, undergraduate students of 17-24 years were recruited for the study if they fall under the succeeding criteria: individuals who have been using a mobile phone to make/receive calls for 2-5 years, individuals who keep their phone attached to the ear while making/receiving a call, and individuals who have been making/receiving calls (or together) for 30 min per day. However, individuals with preexisting ear disorders, a history of sudden loud noise or chronic noise exposure, using headphones for recreational purposes continuously for more than two hours/day, and using headphones for making/receiving calls, with any systemic diseases such as diabetes and hypertension, with a history of taking ototoxic medications in the past; those with a history of measles, mumps, rubella, and jaundice; and those with a history of alcohol abuse were excluded from the study. The entire study was done in two stages: stage 1, questionnaire survey, and stage 2, pure tone audiometry. Stage 1, questionnaire survey: A questionnaire validated by a subject expert for question accuracy was used to obtain the extent of phone usage among participants. They were then grouped as long-term users, intermediate-term users, and short-term users as per the criteria described below. Stage 2, pure tone audiometry: Each group of participants underwent audiometry as per the American Speech-Language-Hearing Association guidelines using a Pure Tone Audiometer (AMBCO-650A, CA, USA).

Stage 1 (questionnaire survey)

Participation was voluntary, and informed consent was obtained from all the subjects. As stated above, a validated questionnaire on mobile phone usage information of participants was used to analyze the extent of mobile phone usage (in particular call duration) among students. The duration of use of mobile phones for making and receiving calls was cross-examined [14]. Following this, those participants who met the above-stated inclusion criteria were grouped and invited to the next stage of the study. The study groups were as follows: group 1, long-term user (a mobile phone user for the past five years and spent 30 min per day making/receiving calls); group 2, intermediate-term user (a mobile phone user for the past four years and spent 30 min per day making/receiving calls); and group 3, short-term user (a mobile phone user for the past three years or less and spent 30 min per day making/receiving calls).

Stage 2 (pure tone audiometry)

Audiometry was done as per American Speech-Language-Hearing Association guidelines [15]. A written informed consent was obtained from all participants before performing audiometry. The test was performed in a quiet room by an expert using a Pure Tone Audiometer (AMBCO-650A, CA, USA). Briefly, a pulsed tone was selected in the audiometer, and the frequency and intensity were set to 250-8000 Hz and 40 dB, respectively. Each participant was instructed to respond to the tone presented by pushing the patient response switch every time a tone was heard. During the test, the headband and receiver were placed on the patient’s ear tightly but comfortably. The red receiver was placed over the right ear and the blue receiver over the left ear. Following this, a tone was presented for 1-2 seconds, and as the participant responded, the intensity lowered to 5 dB decrements. Green light under response indicates sound heard by the participant. The procedure was done for the following frequencies 250, 500, 1000, 2000, 3000, 4000, 6000, 8000 Hz and then back to 250 Hz. It was repeated in the other ear, and an audiogram was plotted for each participant. Hearing function was determined as per the following standard classification: 00-15 dB, normal hearing; 16-25 dB, slight hearing loss; 26-40 dB, mild hearing loss; 41-55 dB, moderate hearing loss; 56-70 dB, moderately severe hearing loss; 71-90 dB, severe hearing loss; and 91 dB and above, profound hearing loss.

Statistical analysis

The questionnaire survey results are reported in percentages. The hearing loss between various groups at different frequencies was represented as mean ± SEM. One-way analysis of variance (ANOVA) and post hoc Tukey’s tests were used to determine the difference between the means of various groups (long term, intermediate term, and short term). However, a two-way ANOVA was used to determine the hearing loss in participants with, right/left ear dominance, 4G/5G users, and in different call durations. A p-value of ≤0.05 was considered statistically significant. GraphPad Prism software version 5.01 (San Diego, CA, USA) was used for data analysis.

## Results

Stage I (questionnaire survey)

Among the 78 participants, 40 (51.28%) were males and 38 (48.71%) were females (Table 1). Sixty-four (82.05%) of the participants responded that the right ear was their dominant ear, and for the remaining 14 (17.95%), it was the left ear. Forty-five participants (57.69%) use a 4G phone, while 32 (41.02%) use a 5G, and only one participant (1.28%) uses a 2G phone. Forty-five participants (57.69%) preferred to use the phone for texting, while 33 (42.30%) used it to make calls. Fifty-four participants (69.23%) firmly attached the phone over their ears while making/receiving a call, but 24 (30.76%) did not. Among the participants, the average call duration was also different. Thirty-one participants (39.74%) have used the phone for making calls for 30 min/day, while 22 (28.20%) participants have used it for one hour/day, 14 (17.94%) used it for two hours/day, and the remaining 11 (14.10%) used it for three hours/day. Fifty-nine participants (75.64%) felt no discomfort after making a call (mean call duration/day; 68.0 ± 50 min). However, 11 (14.10%) participants felt a headache (mean call duration/day; 76.36 ± 55.91 min), seven (8.97%) experienced tinnitus during a call (mean call duration/day; 108.0 ± 60.33 min), and only one (1.28%) felt nausea after making a call. Moreover, only one participant felt a decline in hearing ability after making a call, but the remaining 80 (98.76%) did not feel the same (Table 1).

**Table 1:** Participants' demographics and mobile phone usage information

Participants and their details (n = 78)	
Age (mean ± SD)	20.18 ± 1.52
Male participants	40 (51.28%)
Female participants	38 (48.71%)
Years of mobile phone usage	
5 years	51 (65.38%)
4 years	12 (15.38%)
3 years	9 (11.53%)
2 years	6 (7.69%)
Mobile phone generation is used	
5G phone users	32 (41.02%)
4G phone users	45 (57.69%)
2G phone users	1 (1.28%)
The preferred usage of mobile phones for calling/texting	
Calling	33 (42.30%)
Texting	45 (57.69%)
Dominant ear	
Right	64 (82.05%)
Left	14 (17.95%)
Attaching the phone over the ear during a call	
Yes	54 (69.23%)
No	24 (30.76%)
Call duration per day	
30 min	31 (39.74%)
1 hour	22 (28.20%)
2 hours	14 (17.94%)
3 hours	11 (14.10%)
Discomfort after a call	
No discomfort	59 (75.64%)
Headache	11 (14.10%)
Nausea	1 (1.28%)
Tinnitus	7 (8.97%)
Feeling a decline of hearing ability after calling	
Yes	1 (1.28%)
No	77 (98.71%)

Stage II (pure tone audiometry)

**Figure 1:**
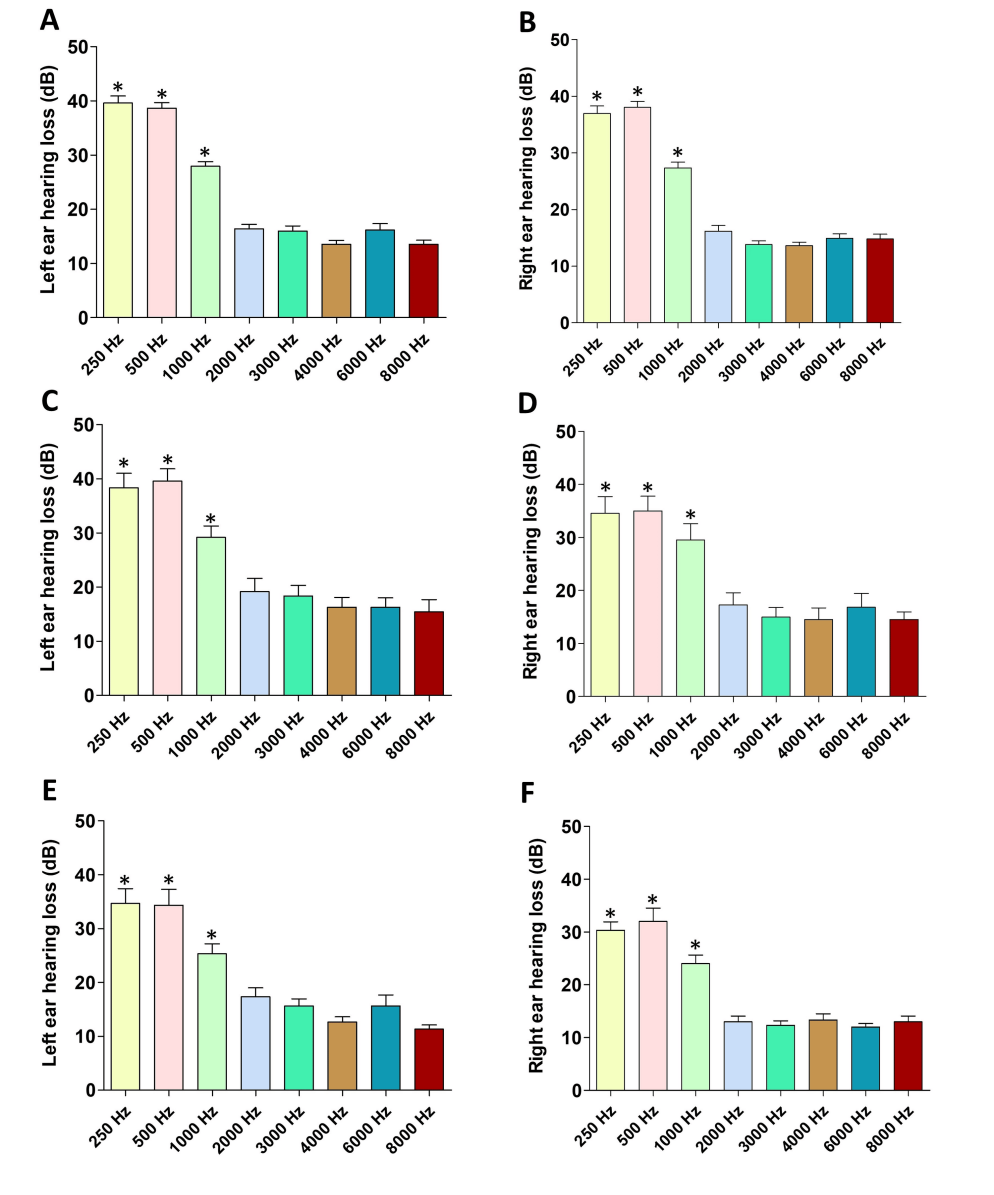
Hearing loss in the left and right ears of long-term (A and B), intermediate-term (C and D), and short-term (E and F) mobile phone users ANOVA: analysis of variance The hearing loss followed a certain pattern in both the right and left ears. Note the significant hearing loss at frequencies 250 Hz, 500 Hz, and 1000 Hz compared to the higher frequencies. *p < 0.05 (250 Hz vs 1000 Hz/2000 Hz/3000 Hz/4000 Hz/6000 Hz/8000 Hz, one-way ANOVA and post hoc Tukey’s tests, *p < 0.05; 500 Hz vs. 1000 Hz/2000 Hz/3000 Hz/4000 Hz/6000 Hz/8000 Hz, one-way ANOVA and post hoc Tukey’s tests, *p < 0.05; 1000 Hz vs. 2000 Hz/3000 Hz/4000 Hz/6000 Hz/8000 Hz, one-way ANOVA and post hoc Tukey’s tests, *p < 0.05)

**Figure 2:**
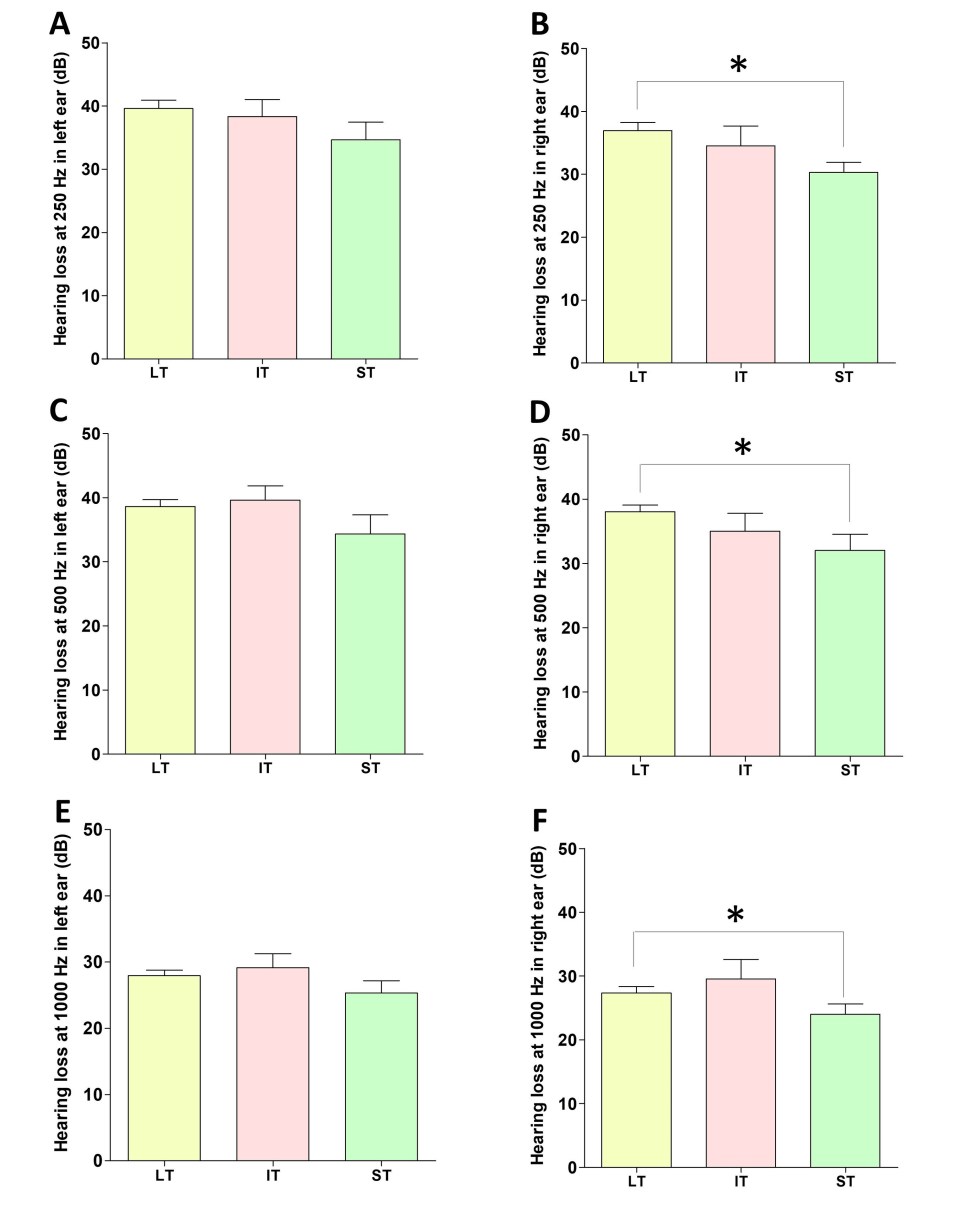
Comparison of hearing loss at 250 Hz (A and B), 500 Hz (C and D), and 1000 Hz (E and F) frequencies at the left and right ears of long-term (LT), intermediate-term (IT), and short-term (ST) mobile phone users ANOVA: analysis of variance *p < 0.05 (Hearing loss at the right ear, LT vs ST, one-way ANOVA and post hoc Tukey’s tests, *p < 0.05)

In the audiometry, mild to moderate hearing loss was evident in the long-term (Figures 1A, 1B), intermediate-term (Figures 1C, 1D), and short-term (Figures 1E, 1F) group participants at frequencies 250 Hz, 500 Hz, and 1000 Hz. However, the hearing loss at higher frequencies (between 2000 Hz and 8000 Hz) was not significantly high compared to the hearing loss at the lower frequencies. This hearing loss pattern was almost similar in participants in both left (Figures 1A, 1C, 1E) and right (Figures 1B, 1D, 1F) ears. Additionally, a comparison of the extent of hearing loss at lower frequencies (250 Hz, 500 Hz, and 1000 Hz) in the right and left ears demonstrated that hearing loss was significant in long-term users compared to short-term users (Figures 2B, 2D, 2F) in the right ear. Although hearing loss was evident in the left ear, there was no significant difference among long-term, intermediate-term, and short-term users in 250 Hz (Figure 2A), 500 Hz (Figure 2C), and 1000 Hz (Figure 2E) frequencies in the left ear. The majority of participants used their right ear while using the phone (Table 1), and hearing loss in their right ear depends on their length of usage (long term/intermediate term/short term) in the right ear.

**Figure 3:**
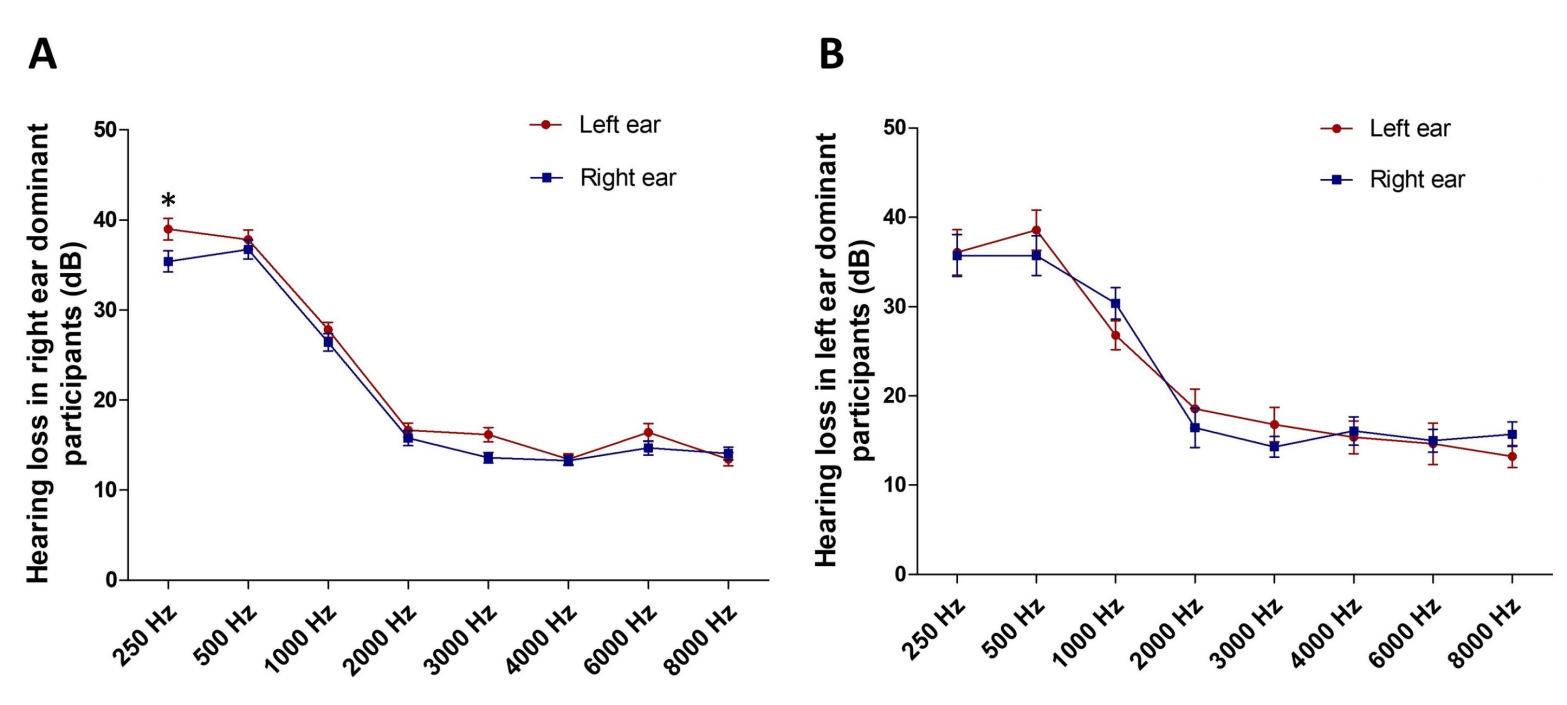
Hearing loss in participants with right (A) and left ear (B) dominance ANOVA: analysis of variance In the right ear dominant participants, the hearing loss was most significant at 250 Hz, 500 Hz, and 1000 Hz compared to other frequencies. Left ear hearing loss was significantly different at 250 Hz (*p < 0.05) compared to the right. The hearing loss was significant at 250 Hz, 500 Hz, and 1000 Hz in left ear dominant participants too, but there was no significant difference observed in the hearing loss values between their right and left ears. *p < 0.05 (Hearing loss in the right ear dominant participants at various frequencies in left vs right ears, two-way ANOVA and post hoc Bonferroni’s tests, *p < 0.05)

Hearing loss in participants with right and left ear dominance was further analyzed. In participants with right ear dominance, the hearing loss was most significant at 250 Hz, 500 Hz, and 1000 Hz compared to other frequencies and was found to be similar in the right and left ears except at 250 Hz (Figure 3A). At 250 Hz, hearing loss in the left ear was 38.98 ± 1.21 dB, but it was 35.39 ± 1.17 dB in the right ear, and this was significantly different (Figure 3A, *p < 0.05). In participants with left ear dominance, the hearing loss was most significant at 250 Hz, 500 Hz, and 1000 Hz compared to other frequencies and was observed to be similar in both ears (Figure 3B).

**Figure 4:**
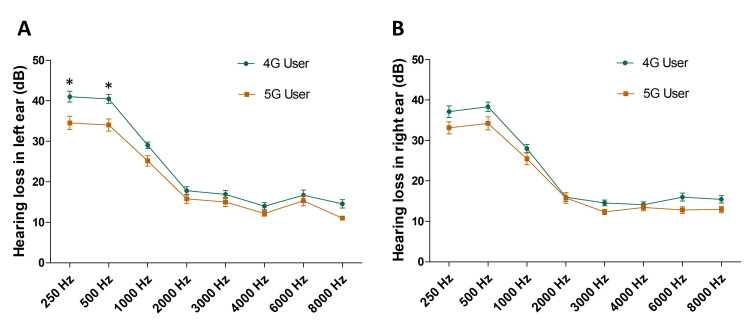
Hearing loss in 4G and 5G phone users in left (A) and right (B) ears ANOVA: analysis of variance Significant hearing loss was found in 4G phone users compared to 5G phone users at 250 Hz and 500 Hz in the left ear but not at other frequencies. However, there was no statistically significant difference in hearing loss between 4G and 5G phone users at any frequency in the right ear (*p < 0.05) (Hearing loss in the left ear at 250 Hz/500 Hz frequencies in 4G vs 5G phone users, two-way ANOVA and post hoc Bonferroni’s tests, *p < 0.05)

Right and left ear hearing loss in 4G and 5G phone users was compared, and it was revealed that in the left ear, significant hearing loss was found in 4G phone users compared to 5G phone users at 250 Hz (*p < 0.05) and 500 Hz (*p < 0.05) (Figure 4A). There were no statistically significant differences in hearing loss in 4G and 5G phone users at frequencies between 1000 Hz and 8000 Hz (Figure 4A). Moreover, there was no statistically significant difference in hearing loss between 4G and 5G phone users at any frequency in their right ear (Figure 4B).

**Figure 5:**
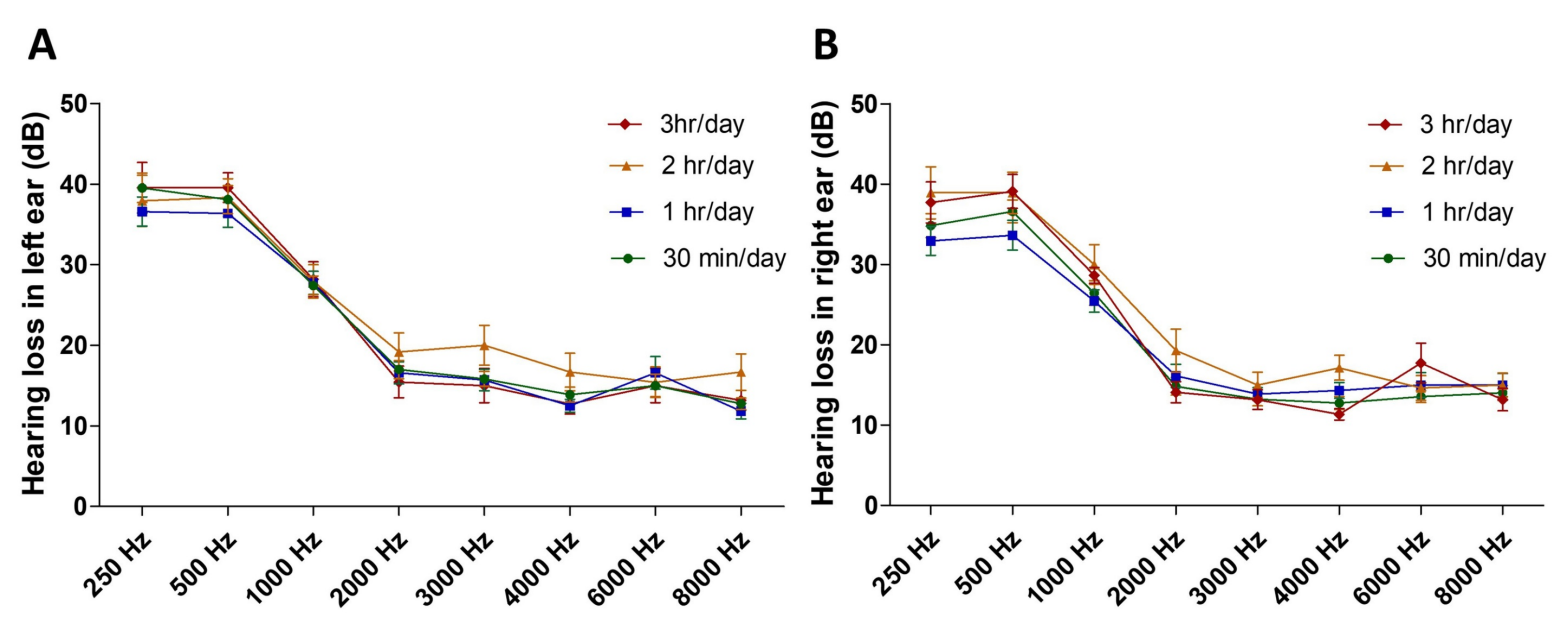
Hearing loss in the left (A) and right (B) ears in participants with different call durations The left and right ear hearing loss in participants with various call durations was not different at various frequencies tested. Note that significant hearing loss was evident at 250 Hz, 500 Hz, and 1000 Hz compared to other frequencies in both ears irrespective of their per-day average call duration

The relationship between hearing loss and call duration was also analyzed. It was found that the left (Figure 5A) and right (Figure 5B) hearing loss in participants with various call durations was not different at various frequencies tested. However, irrespective of their per-day average call duration, the hearing loss was evident at frequencies 250-1000 Hz in both the left (Figure 5A) and right (Figure 5B) ears compared to other frequencies.

## Discussion

Man-made RF-EMR is ubiquitous, and life without these technologies is unimaginable. Potential public health problems due to radiofrequency waves from various emerging and newer versions of mobile phone radiation are a concern. In the current study, only some participants complained of short-term effects like headaches and tinnitus. However, mild to moderate hearing loss was observed in the lower frequencies (250 Hz, 500 Hz, and 1000 Hz) in both ears of all groups studied. Particularly, hearing loss was most evident in individuals who used a phone for five years or more (long term) suggesting a possible connection between the length of mobile phone use and hearing loss in 250 Hz, 500 Hz, and 1000 Hz frequencies. On the other hand, ear dominance was not found to be a significant factor influencing hearing loss because hearing loss was similar in both ears irrespective of the participant's ear dominance.

Although a few studies have been undertaken to assess the hearing status of long-term mobile phone users, the results are contradictory and inconclusive [16,17]. Previous reports observed high-frequency loss and absent distortion product otoacoustic emissions with an increase in the duration of mobile phone use and excessive use of mobile phones [18]. Additionally, users with some complaints during mobile phone use demonstrated absent distortion product otoacoustic emissions and abnormalities in auditory brainstem response [18]. A clinical study by Jadia et al. [19] on the adverse effects of mobile phones on hearing in healthy adults found that participants exposed to mobile phones for more than four hours/day for four years had moderate to severe hearing loss. The current study results corroborate this observation to an extent. Although headaches may be more common in mobile phone users, a prospective study by Auvinen et al. [20] revealed no correlation between mobile phone usage and hearing loss or tinnitus. A study conducted by Benke et al. [21] to determine if exposure to personal music players was a confounder in adolescent hearing health revealed no evidence of confounding by exposure to personal music players.

On the other hand, some research does not suggest an effect of mobile phone radiation on human hearing. An observational study conducted by Sharma et al. [22] found no short-term effect of mobile phones on hearing in young adults, but a long-term influence on hearing with increasing age cannot be ruled out. Bhagat et al. [23] have reported that phone use did not cause subjective symptoms, such as headache, tinnitus, or sensations of burning or warmth behind, around, or on the phone-using ear. They also did not find statistically significant differences in high-frequency pure-tone average between the phone-using ears and the control ears suggesting phone use does not cause hearing loss. Furthermore, a systematic review that analyzed just five relevant studies (two cross-sectional and three cohort studies) found no link between hearing loss and cell phone use [24], which further warrants the need for more research in this field. Panda et al. [18] found no significant change in audiometric parameters in a retrospective case-control study, although they did find a trend of high-frequency hearing loss with increased length of mobile phone use.

However, in the current study, hearing loss was observed in the low frequencies. Moreover, this was associated with their 4G and 5G phone use wherein hearing loss was significant in participants using 4G compared to 5G phones. There could be multiple reasons for hearing loss in participants. It could be due to the RF-EMR exposure from their phones. Mobile phone technology uses RF-EMR from 450 to 3800 MHz, and the very recent 5th generation technology uses a submillimeter (3-30 GHz) or millimeter-wave (30-300 GHz) spectrum. When a person uses a mobile phone, the area of contact of the device including the skin around the ear, the inner ear, the vestibulocochlear nerve, and a small portion of the temporal lobe absorbs all the radiation it emits. Whether this absorption causes health effects such as changes in the functioning of the hair cells, leading to altered hearing mechanisms, is something yet to be established, because, in this study, the hearing ability was reduced in the nondominant ear along with the dominant ear, suggesting other potential mechanisms of hearing loss. The other possibility is that although participants have reported that they use only one ear (the dominant) while calling through mobile phones, in real life, they might be using both ears as this not only depends on preference but also is situation-based. Another possibility is the repeated exposure to sound from a mobile phone causing hearing loss in both ears. However, reports suggest that repeated noise exposure initially affects hearing most at frequencies around 3000-6000 Hz [25]. However, in the current study, lower-frequency hearing is affected compared to higher frequency suggesting mechanisms other than the noise-induced effect is involved in hearing loss. The other possibility for hearing loss that could not be excluded is the cumulative effect of both sound and RF-EMR emitted from a mobile phone. To categorically prove this, further research is needed. 

RF-EMR emitted by a mobile phone is absorbed by the human brain if one closely keeps the phone over the head while talking which in turn increases the brain tissue temperature [26]. This absorption (SAR) depends on several factors, such as the technology used in the phone (1st-5th generation), the distance between the phone and the tissue, the extent (length of call) of use, and the user’s location (or distance) from the mobile phone tower [27]. In the current study, we found that along with average calls per day, participants' number of years of usage also tends to be a factor for their altered hearing ability. This partly corroborates with the results of a recent study that observed measurable changes in auditory brainstem responses (ABRs) in long-term mobile phone users [28]. These observations demonstrate the need to conduct further detailed investigations on long-term mobile phone users including young adults and adults.

There are some limitations of this study that require elaboration. In this study, the dominant ear data was recorded by considering the participant's response, and this was not determined by physiological tests which could be one of the limitations of the study. Moreover, a few candidates may have been exposed to sound pollution due to various reasons. Although this information was asked in the questionnaire and efforts have been made to exclude such participants from the study, this was purely based on the participants' answers which could be a limitation. Another limitation is not obtaining the mobile phone SAR data from the phone users and/or not recording the SAR data using a spectrum analyzer. Although in the current study, we have evaluated the hearing ability among nearly 78 young adults, this sample size might not be a great representation of a population and hence may not be able to generalize the results of the study to an entire population which might be another shortcoming of this research. We couldn’t perform additional tests such as ABR and otoacoustic emissions (OAEs) on the participants to obtain further information about their hearing apparatus functioning which is another limitation of this study. Therefore, a carefully designed large-scale study that considers all these variables and confounding factors will provide a much clearer understanding of RF-EMR effects on human hearing. Since the fastly growing mobile phone technology is surpassing our researching abilities, the need for this immediate research has paramount importance.

## Conclusions

The mobile phone usage caused mild to moderate hearing loss at the following frequencies: 250 Hz, 500 Hz, and 1000 Hz. Hearing loss was evident in individuals who have used their phones for more than 30 min/day for five years continuously compared to individuals who have used their phones 30 min/day for four years and/or 30 min/day for less than three years. This pattern was similar in both the right and left ears. Ear dominance did not play a significant role in influencing hearing loss in participants. However, significant hearing loss was found in 4G phone users compared to 5G phone users at 250 Hz and 500 Hz but not with frequencies between 1000 Hz and 8000 Hz particularly in the left ear. Moreover, irrespective of the participant's per-day average call duration, the hearing loss was evident at frequencies 250-1000 Hz in both the left and right ears compared to other frequencies. Mobile phone usage induced uncomfortable sensations at least in some participants.
